# Spectrum of dominant Charcot-Marie-Tooth disease due to SLC12A6 variants

**DOI:** 10.1136/jnnp-2025-336643

**Published:** 2026-01-07

**Authors:** Christopher J Record, Tiffany Grider, Adriana P Rebelo, Christian Laurini, Mariola Skorupinska, Matt C Danzi, Roy Poh, Pedro J Tomaselli, Rodrigo S Frezatti, Natalia Dominik, Bianca Grosz, Melina Ellis, Kishore R Kumar, Matthew B Harms, Conrad C Weihl, Wilson Marques Júnior, Kristl G Claeys, Julian C Blake, James KL Holt, Astrid Weber, Ryan Jacobson, Richard T Dineen, Yuri M Falzone, Stefano C Previtali, Manoj P Menezes, Steve Vucic, Matilde Laura, Marina L Kennerson, Michael E Shy, Stephan Zuchner, Mary M Reilly

**Affiliations:** 1 Centre for Neuromuscular Diseases, Department of Neuromuscular Diseases, UCL Queen Square Institute of Neurology, London, UK; 2 Department of Neurology, University of Iowa Health Care Medical Center, Iowa City, Iowa, USA; 3 Dr. John T. Macdonald Foundation Department of Human Genetics and John P. Hussman Institute for Human Genomics, University of Miami Miller School of Medicine, Miami, Florida, USA; 4 Neuromuscular Repair Unit, Institute of Experimental Neurology (InSpe), Division of Neuroscience, IRCCS Ospedale San Raffaele, Milan, Italy; 5 Neurogenetics Laboratory, North Thames Genomics Laboratory Hub, National Hospital for Neurology and Neurosurgery, London, UK; 6 Department of Neurology, University of Sao Paulo Ribeirao Preto Medical School, Ribeirao Preto, Brazil; 7 Northcott Neuroscience Laboratory, ANZAC Research Institute, Sydney Local Health District and Faculty of Medicine and Health, The University of Sydney, Sydney, New South Wales, Australia; 8 Molecular Medicine Laboratory and Neurology Department, Concord Repatriation General Hospital, Concord, New South Wales, Australia; 9 Translational Neurogenomics Group, Genomic and Inherited Disease Program, Garvan Institute of Medical Research, Darlinghurst, New South Wales, Australia; 10 Department of Neurology, Columbia University College of Physicians and Surgeons, New York, New York, USA; 11 Department of Neurology, Washington University in Saint Louis School of Medicine, St. Louis, Missouri, USA; 12 Department of Neurology, University Hospitals Leuven, Leuven, Belgium; 13 Laboratory for Muscle Diseases and Neuropathies, KU Leuven, Leuven, Belgium; 14 Department of Clinical Neurophysiology, Norfolk and Norwich University Hospital, Norwich, UK; 15 Walton Centre for Neurology and Neurosurgery, Liverpool, UK; 16 Liverpool Centre for Genomic Medicine, Liverpool Women’s Hospital, Liverpool, UK; 17 Department of Neurological Sciences, Rush University Medical Center, Chicago, Illinois, USA; 18 Vita-Salute San Raffaele University, Milan, Italy; 19 TY Nelson Department of Neurology and Neurosurgery, The Children’s Hospital at Westmead, Westmead, New South Wales, Australia; 20 The Children’s Hospital of Westmead Clinical School, Faculty of Medicine and Health, The University of Sydney, Sydney, New South Wales, Australia; 21 Brain and Nerve Research Centre, Concord Hospital, Concord Clinical School, The University of Sydney, Sydney, New South Wales, Australia

**Keywords:** NEUROPATHY, HMSN (CHARCOT-MARIE-TOOTH), CHANNELS, GENETICS

## Abstract

**Background:**

Heterozygous variants in *SLC12A6* have recently been shown to cause dominant Charcot-Marie-Tooth disease (CMT). We aim to characterise the phenotype of patients with previously reported and novel heterozygous variants in the gene and understand any genotype-phenotype correlation.

**Methods:**

Patients were clinically and genetically assessed in sites from Europe, Australia, Brazil and the USA. All patients underwent whole exome or whole genome sequencing. Variants were classified using American College of Medical Genetics and Genomics criteria.

**Results:**

Twenty-three individuals from 13 families carried nine variants classified either as pathogenic/likely pathogenic or variants of uncertain significance segregating in multiple family members, including five novel variants. Forty-eight percent (11/23) were male with a mean age of disease onset of 15.7 years (range 1–45 years). Clinical phenotype varied dramatically with genotype; Arg207His and Ser647Pro caused a severe childhood-onset, sensory and motor, conduction-slowing neuropathy, whereas Gly552Asp caused a mild, adult-onset, sensory-predominant neuropathy, Thr991Ala an infantile-onset motor neuropathy, and the Met282Lys/Gly286Cys locus a complex, axonal neuropathy.

**Conclusions:**

Heterozygous variants in *SLC12A6* can cause CMT of all clinical phenotypes, severity and age of onset, depending on the genotype. Such phenotypic diversity has not been described for any other CMT gene, and more work is needed to understand disease mechanisms to guide future therapeutic options.

**WHAT IS ALREADY KNOWN ON THIS TOPIC**

- Heterozygous variants in *SLC12A6* have recently been reported to cause Charcot-Marie-Tooth disease (CMT) without the classical features of the recessive syndrome, the latter causing a complex neuropathy with agenesis of the corpus callosum and a neurodevelopmental deficit.

**WHAT THIS STUDY ADDS**

- Our detailed genotype-phenotype study clarifies the breadth of peripheral neuropathy syndromes associated with heterozygous variants in *SLC12A6*. This breadth of phenotype has not been reported previously in a single CMT gene. The spectrum ranges from infantile-onset, severe, conduction-slowing neuropathy (CMT1, CMTi) to adult-onset sensory-predominant mild disease (hereditary sensory neuropathy, CMT2), as well as pure motor (hereditary motor neuropathy) and more complex neuropathies. There is a distinct, variant-specific genotype-phenotype correlation.

**HOW THIS STUDY MIGHT AFFECT RESEARCH, PRACTICE OR POLICY**

- *SLC12A6* variants should be considered in all cases of suspected inherited neuropathy and included in the heterozygous state on all neuropathy gene panels. More work is needed to understand why these variants cause neuropathy and why there is such a breadth of phenotype which is seemingly genotype specific.

## Introduction

 Charcot-Marie-Tooth disease (CMT) encompasses a heterogeneous clinical-genetic spectrum including multiple clinical subtypes (CMT1, demyelinating, upper limb motor nerve conduction velocities (MNCV) <38 m/s; CMT2, axonal, MNCV >38 m/s; CMTi, intermediate which overlaps with CMT1 and 2, MNCV <45 m/s but >25 m/s; hereditary sensory neuropathy (HSN), pure sensory neuropathy; hereditary motor neuropathy (HMN), pure motor neuropathy) and a rapidly expanding genetic basis in variation of more than 100 genes.

Andermann syndrome, first clinically defined in the 1970s as a complex syndrome of inherited peripheral neuropathy, the then so-called ‘mental retardation’, with or without agenesis of the corpus callosum,^1^ was shown in 2002 to be caused by recessive variants in the gene *SLC12A6,* encoding the potassium-chloride co-transporter protein KCC3. This is most commonly found in French Canadians due to the c.2436delG founder mutation.^2^ The severe syndrome is not part of the classical CMT umbrella due to significant multisystem involvement and would be considered a complex inherited neuropathy.^3^ However, several disease-genes have been shown to demonstrate both autosomal dominant and recessive patterns of inheritance, often yielding different syndromes, including the aminoacyl tRNA synthetase group of genes.^4^ A single case of a heterozygous variant in *SLC12A6* causing isolated neuropathy was first identified in 2016,^5^ and recently studies have presented recurrent variants in the gene causing dominant CMT.^6^,^9^ We present the largest cohort of patients with heterozygous variants in *SLC12A6* and discuss the striking phenotypic variability and implications for further research and clinical practice.

## Methods

Families with previously genetically undetermined CMT were recruited from sites in Australia, Belgium, Brazil, Italy, the UK and the USA. Patients were clinically assessed by neuromuscular/neurogenetic experts, using the validated CMT Examination and Neuropathy Scores (CMTES/NS).^10^ All patients with CMT1 or CMTi previously tested negative for the *PMP22* duplication. Genetic testing was performed with either whole exome sequencing (WES) or whole genome sequencing (WGS).^11^ Virtual panels were applied to WES/WGS data to exclude known causes of neuropathy. Variants were classified according to American College of Medical Genetics and Genomics (ACMG) criteria with additional best practice guidelines from the Association for Clinical Genomic Service recommendations.^12^,^14^

## Results

**Figure 1:**
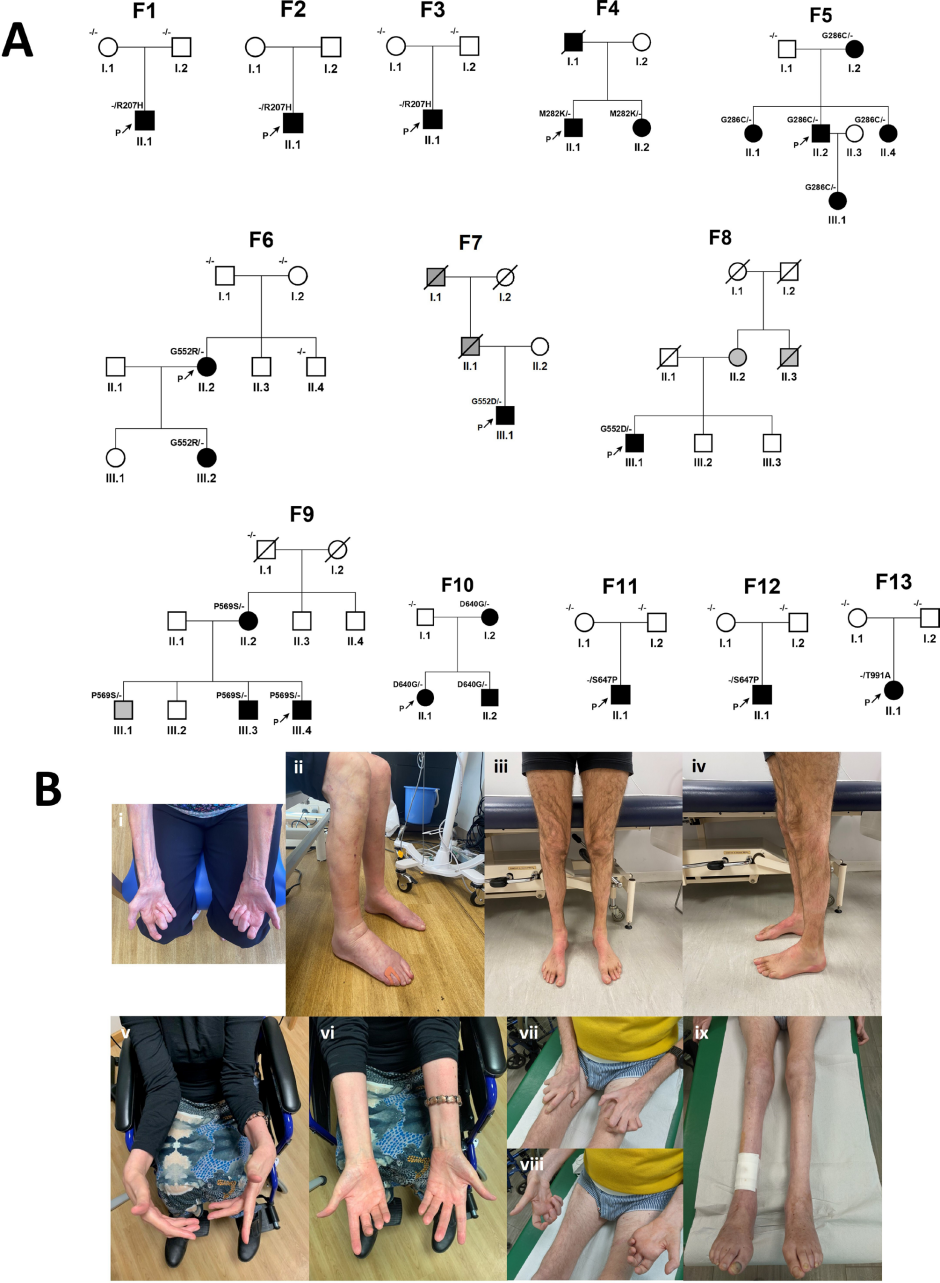
Pedigrees and clinical photographs. (A) Pedigrees of the 13 families with pathogenic or likely pathogenic variants in *SLC12A6* or variants of uncertain significance segregating in >1 individual. (B) Images showing varying degrees of severity of muscle atrophy, distal limb contracture and use of mobility aids.

**Figure 2:**
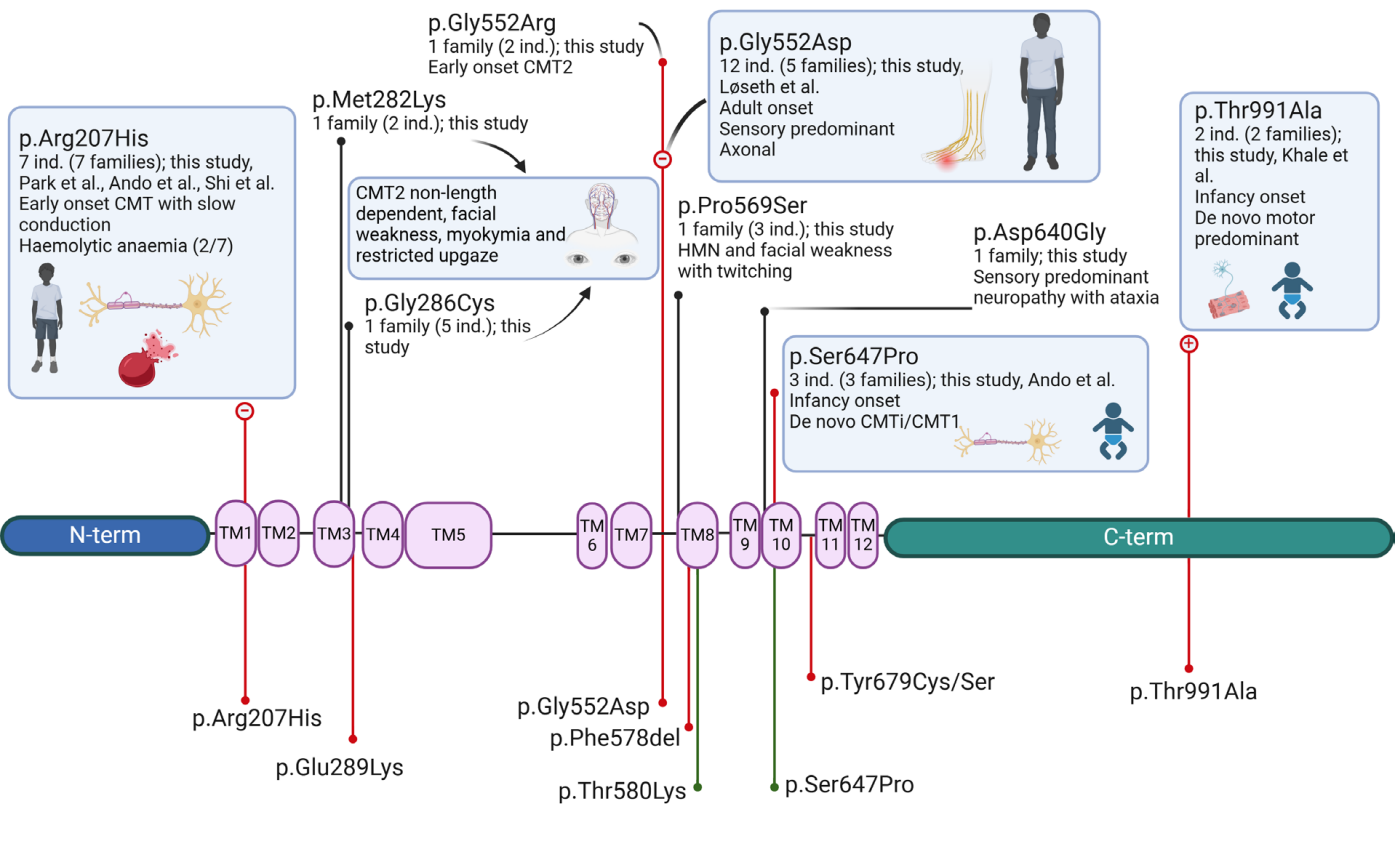
Genotype-phenotype correlation schematic of protein structure based on the Cryo-EM structure of SLC12A6 (KCC3) demonstrated by Chi *et al*^29^ after amino acid positions converted to the MANE transcript. Previously reported heterozygous variants below the protein; pathogenic variants denoted with red line, variants of uncertain significance (VUS) with green line. Variants reported in this study above the protein (VUS with single individual not shown); pathogenic variants denoted with red line, VUS with more than one affected individual with black line. Boxed descriptions indicate more than one family exhibiting the same phenotype at a particular locus. Encircled+ and – denote functionally demonstrated gain-of-function and loss-of-function variants respectively. AA, amino acid; CMT, Charcot-Marie-Tooth disease; Ind., individuals; Term, terminus; TM, transmembrane. Figure created with BioRender.com.

Twenty-seven individuals with CMT from 17 families were identified with heterozygous variants in *SLC12A6*. Of these, four individuals from four families carried four novel variants of uncertain significance (VUS) and were either sporadic cases or had a dominant family history where segregation could not be performed. These were considered ‘cold’ VUSs and are therefore not discussed in detail in this study (see online supplemental table 1). The remaining 23 individuals from 13 families carried nine variants that were either recurrent pathogenic (or likely pathogenic) variants or VUS segregating in multiple affected family members and believed to be disease causing (table 1, figures1  2). Forty-eight percent (11/23) were male with a mean (median) age of disease onset of 15.7 (12.5) years (range 1.0–45 years; where an age range was reported for the disease onset, for the purposes of calculating averages the midpoint was taken, and infancy was arbitrarily assigned 1.5 years). The mean (median) age at assessment was 40.2 (41.5) years, range 7.5–76 years. Fifty-five per cent (11/20) had pes cavus or high arches. Phenotypes varied dramatically across the cohort according to genotype and covered most of the described clinical spectrum of CMT; infantile, childhood and adult onset; and CMT1, CMTi, CMT2 (including sensory predominant and more complex neuropathy syndromes) and motor-predominant CMT (table 2 (summary), figure 2, online supplemental table 2 (in full)). Mild neurodevelopmental symptoms were present in only one individual (F11). An MRI brain was performed in 10/23 (43%) which was reported either normal (eight) or with small areas of non-specific signal change (two). No corpus callosum abnormalities were identified. Neurophysiology is detailed in brief in table 3 (see online supplemental table 3 for full-length table) and discussed by variant below.

**Table 1:** Variant classification

Variant nucleotide	c.620G>A	c.845T>A	c.856G>T	c.1654G>C	c.1655G>A	c.1705C>T	c.1919A>G	c.1939T>C	c.2971A>G
Variant amino acid	p.Arg207His	p.Met282Lys	p.Gly286Cys	p.Gly552Arg	p.Gly552Asp	p.Pro569Ser	p.Asp640Gly	p.Ser647Pro	p.Thr991Ala
Hg19	15:34549913:C:T	15:34547494:A:T	15:34547483:C:A	15:34538064:C:G	15:34538063:C:T	15:34538013:G:A	15:34537510:T:C	15:34537490:A:G	15:34528980:T:C
Hg38	15:34257712:C:T	15:34255293:A:T	15:34255282:C:A	15:34245863:C:G	15:34245862:C:T	15:34245812:G:A	15:34245309:T:C	15:34245289:A:G	15:34236779:T:C
ACMG classification	Pathogenic	VUS	VUS	Likely pathogenic	Pathogenic	VUS	VUS	Likely pathogenic	Likely pathogenic
Criteria used	PP3, PM2, PM6_strong, PS4_mod, PS3_mod	PP3, PM2	PP3, PM2, PP1_mod	PP3, PM2, PM5, PM6_supp	PP3, PM2, PP1_strong, PS4_mod, PS3_mod	PP3	PP3, PM2	PP3, PM2, PM6, PS4_mod	PS4_supp, PM2, PM6, PS3_mod, PP2
AC GnomadV2/V3/V4 (frequency)	0/0/0	0/0/0	0/0/0	0/0/0	0/0/0	0/0/9 (5.6e-6)	0/0/0	0/0/0	0/0/0
Amino acid residue conservation (phyloP100way)	Zebrafish (7.80)	Zebrafish (9.22)	Zebrafish (7.87)	Zebrafish (7.21)	Zebrafish (7.54)	Zebrafish (9.60)	Zebrafish (7.93)	Zebrafish (4.88)	Zebrafish (7.95)
REVEL/CADD	0.957/33	0.964/29.9	0.963/32	0.959/28.8	0.976/27.6	0.892/28.2	0.983/32	0.939/26.1	0.397/27.1
Total families	7	1	1	1	7	1	1	3	2
Total individuals	7	2	5	2	12	3	3	3	2
Previously reported	Ando *et al* (one family), Shi *et al* (one family), Park *et al* (two families)	Novel	Novel	Novel	Loseth *et al* (five families)	Novel	Novel	Ando *et al* (one family)	Kahle *et al* (one family)
Segregation (N)	–	1/2	1/16	1/2	1/32	1/4	1/4	–	–
De novo data	De novo without paternity x5 (Ando *et al* x1, Park *et al* x2, this study x2)	–	–	De novo without paternity x1 (this study)	–	–	–	De novo without paternity x2 (this study)	De novo without paternity x2 (this study, Kahle *et al*)
Functional evidence	Oocytes injected with mutant mouse RNA – complete absence of K+ flux in hypotonic conditions (Park *et al*)	–	–	–	Oocytes expressed with mutant – complete absence of K+ flux in hypotonic conditions (Loseth *et al*)	–	–	–	T991A knock-in mice develop neuropathy and mutant fibroblasts exhibit exaggerated K+ flux in isotonic conditions (Kahle *et al*)

All variants are reported in MANE transcript ENST00000354181.8.

N = probability of observed co-segregation occurring by chance as defined by the number of informative meioses, totalled over all families.

PM6 de novo data are assigned using ClinGen Sequence Variant Interpretation Recommendation for de novo Criteria (PS2/PM6) - Version 1.1 Working Group Page: https://clinicalgenome.org/working-groups/sequence-variant-interpretation/. De novo without paternity – de novo variant where paternity and maternity is not confirmed through genetic testing. PP2 for missense constraint assigned based on constraint value <0.4 (https://www.deciphergenomics.org/gene/SLC12A6/browser accessed 16 April 2025). PP3 assigned if REVEL (https://sites.google.com/site/revelgenomics/about accessed 16 April 2025) >0.7.

AC, allele count; P/LP, pathogenic/likely pathogenic; REVEL, rare exome variant ensemble learner; VUS, variant of uncertain significance.

**Table 2:** Summary of clinical features

Family	Individual	Variant	Inheritance	Phenotype	AAO (years)	AAA (years)	CMTES/NS	Presenting symptom	LL power (dist/prox)	UL power (dist/prox)	Sensory signs	Facial weakness	Other cranial nerve signs
**F1**	II.1	R207H	De novo	CMT1	First decade	20s	18/25	DW	0/4	3/4	Yes	Yes	No
**F2**	II.1	R207H	Sporadic	CMT2	2–3	20s	nd	Falls	1,4/5	4/5	Yes	No	No
**F3**	II.1	R207H	De novo	CMTi	First decade	20s	nd	Weakness	1/4	1/5	Yes	No	Esotropia
**F4**	II.1	M282K	AD	CMT*	Early teens	60s	26/34	DW	0/1	0/3	Yes	Yes+myokymia	RU
	II.2			CMT2	Early teens	60s	19/27	DW	0/4	2/4	Yes	Myokymia	RU, temporalis wasting, ptosis
**F5**	I:2	G286C	AD	CMT2†	40s	70s	21/28	Tripping	0/4	0/5	Yes	No	No
	II:1			CMTi	25	50s	15/21	Slow walking	1/5	4/5	Yes	Yes+myokymia	RU
	II:2			CMT2	First decade	40s	9/nd	Tripping	2/5	4/5	Yes	Myokymia	No
	II:4			CMT2	20	40s	6/12	Hand cramps	4/5	4/5	Yes	No	No
	III:1			CMT2†	13	Teens	5/nd	DW	4/5	4/5	Yes	No	No
**F6**	II.2	G552R	AD	CMTi	10	40s	24/36	DW/Falls	0/4	1/5	Yes	Yes	No
	III.2			CMTi	5	First decade	nd	DW/Falls	4/5	5/5	No	No	No
**F7**	III.1	G552D	AD‡	HSN	Early 20s	50s	6/10	Balance	5/5	5/5	Yes	No	No
**F8**	III.1	G552D	AD‡	CMT2	40	50s	nd	DW	4/5	4/5	Yes	No	No
**F9**	II.2	P569S	AD	HMN§	34	40s	nd	DW	2-3/4	5/5	No	Myokymia	No
	III.4			HMN	10	40s	8/8	DW	1/4	5/5	No	Yes	No
**F10**	I.2	D640G	AD	CMT2-SP	20s	70s	nd	Unsteadiness	4/5	4/5	Yes	No	Ptosis
	II.1			CMT2-SP	30s	30s	nd	Unsteadiness	5/5	5/5	Yes	No	No
	II.2			CMT2-SP	Teens	30s	nd	Unsteadiness	4/5	5/5	Yes	No	No
**F11**	II.1	S647P	De novo	CMTi	Infancy	Teens	10/17	DW/falls	0/5	2/5	No	Yes	RU, ptosis
**F12**	II.1	S647P	De novo	CMTi¶	Infancy	40s	23/30	DW/falls	0/4	1/4	Yes	No	B/l VC palsy, tongue atrophy
**F13**	II.1	T991A	De novo	HMN	1	First Decade	nd	Delayed motor skills	1/5	4/4	No	No	No

LL (=lower limb) power written as Medical Research Council grade out of 5 dist (=distal)/prox (=proximal), where there is different in distal anterior/posterior compartments a comma separates the values.

UL (= upper limb) power as per lower limb.

* Entirely absent action potentials.

† Very limited study.

‡ Segregation not performed, by history.

§ Neurophysiology not seen.

¶ Low CMAP amplitudes make definitive evaluation of phenotype problematic.

AAA, age at assessment; AAO, age at onset; AD, autosomal dominant; b/l, bilateral; CMAP, compound motor action potential; CMT, Charcot-Marie-Tooth disease; CMTES/NS, CMT Examination Score/Neuropathy Score; DW, difficulty walking; nd, not done; RU, restricted up-gaze; SP, sensory predominant; VC, vocal cord.

**Table 3:** Summary of neurophysiology

Family-individual	Variant	Phenotype	Age at study (years)	Median CMAP mV	Median CV m/s	Peronel (TA) CMAP mV	Peronel (TA) CV m/s	Median SNAP µV	Sural SNAP µV	EMG
F1-II.1	R207H	CMT1	20s	1.1	27	Absent	Absent	Absent	Absent	ND
F2-II.1	R207H	CMT2	Teens	2.9	41	1.4	39	1.5	Absent	Chronic denervation
F3-II.1	R207H	CMTi	20s	2	27	0.1	31	Absent	Absent	Severe LD chronic denervation
F4-II.1	M282K	CMT*	60s	Absent	Absent	Absent	Absent	Absent	Absent	Severe LD chronic denervation
F4-II.2		CMT2	60s	2.6	44	Absent	Absent	Absent	ND	Distal and proximal denervation
F5-I:2	G286C	CMT2†	70s	Absent	Absent	ND	ND	Absent	ND	ND
F5-II:1		CMTi	50s	2	33	0.3	46	Absent	Absent	Severe distal and proximal denervation
F5-II:2		CMT2	40s	1.2	43	0.8	41	Absent	Absent	LD chronic denervation+acute denervation
F5-II:4		CMT2	40s	7.1	49	3.1	42	Absent	Absent	Severe distal and proximal denervation
F5-III:1		CMT2†	Teens	ND	ND	ND	ND	Absent	ND	Distal denervation
F6-II.2	G552R	CMTi	40s	Absent	Absent	0.4	25	Absent	Absent	Severe chronic denervation distal>proximal
F6-III.2		CMTi	First decade	3.2	40	ND	ND	Absent	ND	Chronic denervation
F7-III.1	G552D	HSN	50s	7.3	45	3.7	35	Absent	Absent	Normal
F8-III.1	G552D	CMT2	50s	1.6	44	2.1	39	Absent	Absent	Chronic denervation
F9-III.4	P569S	HMN	40s	7.5	61	Absent	Absent	12.1	10.8	Widespread acute and chronic denervation+CRD
F10-I.2	D640G	CMT2-SP	70s	4.2	50	2.4	44	Absent	Absent	ND
F10-II.1		CMT2-SP	30s	10.7	50	ND	ND	Absent	Absent	Mild distal denervation in LL
F10-II.2		CMT2-SP	30s	3.7	45	3.2	39	Absent	Absent	Moderate/severe distal denervation in LL
F11-II.1	S647P	CMTi	First decade	2.2	35	ND	ND	Absent	Absent	LD acute and chronic denervation
F12-II.1	S647P	CMTi‡	40s	0.7	25	Absent	Absent	Absent	Absent	LD severe acute and chronic denervation
F13-II.1	T991A	HMN	First decade	2	28	ND	ND	22	17.9	ND

* Entirely absent action potentials.

† Very limited study.

‡ Low CMAP amplitudes make definitive evaluation of phenotype problematic.

CMAP, compound motor action potential; CRD, complex repetitive discharges; CV, conduction velocity; EMG, electromyography,; HMN, hereditary motor neuropathy; HSN, hereditary sensory neuropathy; LD, length-dependant; LL, lower limbs; ND, not done; SNAP, sensory nerve action potential; TA, tibialis anterior.

The nine variants consisted of four previously reported variants c.620G>A p.(Arg207His) (pathogenic), c.1655G>A p.(Gly552Asp) (pathogenic), c.1939T>C p.(Ser647Pro) (likely pathogenic) and c.2971A>G p.(Thr991Ala) (likely pathogenic) and five novel variants c.1654G>C p.(Gly552Arg) (likely pathogenic), c.845T>A p.(Met282Lys), c.856G>T p.(Gly286Cys), c.1705C>T p.(Pro569Ser) and c.1919A>G p.(Asp640Gly) (all VUS, table 1 and figure 2). The four novel VUS not discussed are c.2437–2A>G, c.630G>T p.(Trp210Cys), c.1445C>T p.(Thr482Ile) and c.1217_1218delGGinsTC p.(Trp406Phe) (online supplemental table 1).

Three sporadic cases (F1-3) were found to carry the pathogenic Arg207His, of which two were proven de novo.^6^,^8^ The phenotype was consistently a moderate-severe sensory and motor neuropathy, with onset in the first decade, and conduction slowing predominantly in the intermediate or demyelinating range. Two families were found to carry segregating variants in a novel hotspot containing two variants four amino acids apart (Met282Gly, F4, and Gly286Cys, F5). The phenotype was CMT2 with variable age of onset (range first decade of life to 40s) and severity; the CMTES/NS ranged from very severe (26/34, aged 64) to mild (5/not done, aged 17). Notably, four individuals had facial myokymia (accompanied by facial weakness in 2/4), three had restricted vertical gaze and one had ptosis. Two had a postural tremor and two had plantar flexion weakness preceding ankle dorsiflexion weakness. In some cases, the denervation on EMG was not length dependent.

Two individuals from two families with an autosomal dominant family history (F7 and F8) were found to carry the pathogenic Gly552Asp.^9^ Both presented with difficulty walking in adulthood and had a mild neuropathy classified as HSN (F7) and sensory-predominant CMT2 (F8), respectively. A further family (F6) with childhood-onset sensorimotor neuropathy carried Gly552Arg, a novel change at the same amino acid residue. The variant occurred de novo in the proband, who, when assessed in their 40s, had a severe neuropathy with significant conduction slowing. Two individuals (F11, F12) carried the likely pathogenic variant Ser647Pro, proven de novo in both cases.^7^ Both had moderate to severe, infantile onset neuropathy with significant conduction slowing (table 3, online supplemental table 3). F11 had facial weakness, restricted up-gaze and ptosis (as seen with Met282Lys/Gly286Cys) with history of febrile seizures, speech delay and ADHD, and F12 had bilateral vocal cord palsy, tongue atrophy and dysmorphic facies. The de novo likely pathogenic variant Thr991Ala was found in one individual (F13) with infantile onset motor neuropathy.^5^

Three affected members of family F9 carry the novel variant Pro569Ser. Their phenotype is HMN with some non-length dependent features, with variable age of onset (range 10–34 years). The proband’s sibling (III.3) is clinically affected and has previously been assessed by the authors but clinical information is not available. Individual II.2 had facial myokymia as seen with Met282Lys/Gly286Cys. The proband’s oldest sibling III.1 carries the Pro569Ser variant and in their 50s is reportedly unaffected, although they have not been assessed, which raises the possibility of incomplete penetrance (figure 1A). One family (F10) has three affected members with a sensory predominant CMT2 with ataxia, carrying the novel Asp640Gly.

## Discussion

The families in our study, the largest case series of dominant CMT due to *SLC12A6* variants, contribute to the existing indicators that the clinical spectrum is strikingly broad and appears to have specific genotype-phenotype correlations (figure 2).

### Arg207His causes a childhood-onset, severe conduction slowing neuropathy.

Including the three cases in this study, this variant has been reported in seven sporadic cases from seven families and confirmed to have arisen de novo in 5 of 7.^6^,^8^ It has been detected in patients from a broad range of ancestries including white British, white American, Brazilian, German, Chinese and Japanese. The mean (median) age at symptom onset was 6.2 (2.5) years, with only one outlying case in the literature with onset at 27 years. The majority of patients had significant conduction slowing with mean (median) median nerve MNCV of 33.1 m/s (32 m/s) ranging from 27 to 41 m/s and would be classified as CMTi or CMT1. Interestingly, haemolytic anaemia has been identified in two individuals carrying the Arg207His variant (this study and Park *et al*)^6^ and also Tyr679Ser.^7^ Given the expression of KCC3 in erythrocytes and its importance in cell volume homeostasis, it is likely that haemolytic anaemia is a haematological manifestation of the syndrome, although it remains unclear if this is also genotype specific.^15 16^

### The Met282Lys/Gly286Cys locus causes a complex axonal neuropathy with variable severity.

Our two families at the Met282Lys/Gly286Cys locus show a variable severity, predominantly axonal neuropathy, but presented with the unusual finding of restricted up-gaze and facial myokymia. The age at symptom onset was variable and not all individuals manifested the facial findings.

### Gly552Asp causes a mild, adult-onset sensory-predominant neuropathy.

The phenotype in our cases corroborates work by Løseth *et al* who published 10 affected individuals from five Norwegian families all carrying Gly552Asp.^9^ The predominant phenotype seen in the published cases was a mild, adult-onset, large fibre, sensory-predominant neuropathy. Across the 12 affected individuals from seven families, where applicable, mean (median) age of symptom onset was 30.5 years (31 years). In this previous study, two probands retrospectively had symptoms dating back to their first decade of life (falling more than peers, numbness and unbalance in their legs respectively), but only sought medical opinion in their twenties and beyond. Contrastingly, in the same study, the father of one of the probands developed symptoms in his mid-60s, and two individuals had no symptoms of neuropathy but clinical signs and neurophysiology in keeping with the same sensory neuropathy of their proband sibling. This suggests that *SLC12A6* variants can cause a subclinical neuropathy, and as noted by Løseth *et al*, the Gly552Asp variant could be considered a hypomorphic (ie, with incomplete penetrance) allele. Six of 12 patients had demonstrable weakness, which when present was mild, affirming the sensory predominance. Abnormalities in vibration were the most common clinical finding indicating large fibre deficits, and ataxia was often present. Interestingly, although only seen in one family of two affected individuals in our study, the novel substitution at the same amino acid Gly552Arg causes a more classical sensory and motor neuropathy without the sensory predominance, with childhood onset. The family carrying the novel Asp640Gly all have a similar phenotype to the Gly552Asp with sensory ataxia and variable motor involvement.

### Ser647Pro causes a severe, infantile-onset conduction slowing neuropathy.

We report two individuals with the proven de novo variant Ser647Pro, which combined with the case from Ando *et al* (de novo likely but not proven)^7^ totals three individuals with a near identical phenotype. Motor deficits were noticed at the age of 3 years or below in all cases, and when assessed, all had severe length-dependent weakness of upper and lower limbs. Neurophysiology consistently showed prominent conduction slowing (upper limb MNCV 13–35 m/s) classified as CMT1 or CMTi, although significantly reduced compound motor action potentials (CMAPs) make interpretation of velocities challenging. The cranial nerve and CNS features seen in our cases illustrate that this variant can cause a more complex neuropathy. An important additional point can be made about the reporting of rare diseases via the Ser647Pro variant. Ando *et al* could only classify it as a VUS as a single case with a novel variant. However, their reporting of a VUS in the supplementary material along with detailed clinical phenotype allowed us to upgrade the variant to likely pathogenic. This highlights the importance of literature reporting of novel variants with accompanying phenotype information, even with uncertain disease association; without this information, our variant would have remained a VUS. A similar argument could be made for clinical service laboratories reporting VUS on genetic reports, although the counter-argument can be made for misinterpretation by clinicians and patients, so careful consideration for variant reporting is required.^17 18^ Alternatively, the freely accessible public archive ClinVar (https://www.ncbi.nlm.nih.gov/clinvar/ accessed 15 April 2025) can be used to report variants, with supporting evidence, allowing laboratories to communicate VUS with the wider community.

### Thr991Ala causes an infantile-onset motor neuropathy.

The first case of dominantly inherited *SLC12A6* reported in 2016 by Kahle *et al* was a de novo case of Thr991Ala.^5^ We report a second case of this variant, also found de novo. As with other variants, the phenotype at this locus is near identical in the two reported individuals and contrasts with that seen in any other variant. Both presented with motor deficits by the age of 1 year and when assessed had a pure motor neuropathy, without additional features. This was confirmed by neurophysiology; a motor neuropathy was demonstrated, and although conduction slowing was seen in both cases, this was in the context of significant reduction in CMAPs, making it difficult to draw conclusions about the mechanism of the reduction in velocities. We also acknowledge that both children were examined before the age of 10 years, and a pure motor syndrome seen at this age may progress to include sensory involvement over time.

### Disease mechanisms

Phenotypic overlap between the neuropathy syndrome seen in the heterozygous state as described above, and Andermann syndrome, or agenesis of the corpus callosum with peripheral neuropathy (ACCPN) seen with biallelic variants, is unsurprising. A clinical study and review of ACCPN describe a sensory and motor neuropathy with mean median MNCV of 23–34 m/s. Nerve biopsies showed mixed axonal and demyelinating features, and in some cases suggested a swollen axoplasm. Additional features of ptosis, upgaze palsy and facial weakness as reported in our study were seen in up to 50% of ACCPN.^19^ Myokymia has not been reported in ACCPN but has been seen in dogs with biallelic *SLC12A6* variants.^20^ Null mice show a neuropathic phenotype with thinly myelinated axons, decompaction of myelin, axonal swelling and fibre degeneration on sciatic nerve histology, analogous to the pathology seen in humans. The heterozygous mouse did not develop a neuropathy phenotype, but did have significantly altered exploratory behaviour compared with wild type, hinting that haploinsufficiency in some circumstances may cause a phenotype.^2^

The mechanism by which variants in *SLC12A6* cause disease is not understood. The potassium-chloride co-transporter 3 (KCC3) is part of a homologous family of ion co-transporters (KCC1-4) with structural conservation across the family and molecular conservation across species. The maintenance of potassium and chloride homoeostasis by the KCCs is critical in many physiological processes including cell volume regulation, intracellular chloride concentration and neuronal excitability.^21^ KCC3 is expressed in the central and peripheral nervous system, although the KCC3 transcript has only been detected in juvenile but not adult mouse dorsal root ganglia, Schwann cells and sciatic nerves, indicating its importance in peripheral nerve development.^22 23^ KCC3 shares a structure consisting of 12 transmembrane domains, with an extracellular loop between transmembrane helix 5 (TM5) and 6, and a long intracellular C-terminal domain (CTD) after TM12, with the three other KCCs (figure 2).^24^ The protein structure, and its dimeric form in vivo, is essential for attempting to elucidate specific disease mechanisms and phenotype variability demonstrated herein.

Functional studies for variants causing ACCPN have highlighted the functional importance of the CTD. Although in oocytes the French-Canadian founder mutation c.2436delG p.(Thr813fsX813) within the CTD did not alter trafficking, producing a truncated protein at the cell membrane,^2^ very distal truncation (p.Arg1134Ter) resulted in no K^+^ flux, and intracellularly retained protein, suggesting more distal defects do affect trafficking to the cell membrane.^25^ The biallelic Arg207Cys reported to cause a milder ACCPN syndrome has also been shown to impair transport of the protein to the cell membrane in both oocytes, mammalian cells and human brain tissue, but potentially through a different mechanism to the truncating variants. Full-length, intracellular KCC3 was isolated in the dimeric form, indicating that Arg207Cys does not result in truncated protein and favours formation of homodimeric intracellular structures, impairing protein trafficking.^25 26^

A dominant negative effect of certain KCC3 mutants was first considered by Ding *et al.*^27^ They fortuitously discovered a variant Glu289Gly, at that time not reported to cause disease, that failed to demonstrate K^+^ transport in oocytes. They showed impaired protein trafficking of not only KCC3, but also KCC2 and wild-type KCC3, thus supporting a dominant negative effect of the variant. Before the identification of heterozygous *SLC12A6* variants causing disease, the authors postulated that single non-truncating mutations acting in this way may lead to a different phenotype to loss-of-function variants.^27^ The first heterozygous report of disease due to *SLC12A6* was reported 3 years later.^5^ Subsequently, a heterozygous variant at the Glu289 residue, as predicted by Ding *et al*, was detected in two unrelated Japanese patients with CMT (Glu289Lys), although this was not studied functionally.^7^

High-resolution cryogenic electron microscopy (cryo-EM) has contributed significantly to understanding the structure of the potassium-chloride co-transporter family of proteins and shedding light on potential disease mechanisms. Previous cryo-EM studies on both the Arg207 and Glu289 residues have highlighted the importance of an Arg207-Glu289 salt bridge on transporter activity.^24 28^ Cryo-EM of KCC3 has identified other important sites for ion binding including Thr497 (reported as Thr446 in non-MANE transcript; converted to the MANE transcript ENST00000354181.8) and Tyr283 (Tyr232) for K^+^ binding.^29^ The Met282Lys and Gly286Cys variants identified in this study could potentially be affecting K+ binding either side of the Tyr283 residue. The importance of the Tyr283 and Thr497 residues for K+ coordination was confirmed by Lui *et al* who mutated the mouse KCC3 orthologue at these loci, resulting in complete loss of transporter activity.^30^

Alternatively, a gain-of-function mechanism is presented by Kahle *et al* for the first reported heterozygous variant in *SLC12A6* causing a neuropathy. They showed that Thr991Ala abolished WNK kinase–dependent inhibitory phosphorylation at this residue, with inappropriate transporter activity even in isotonic conditions.^5^ This supports prior work indicating that the Thr991 (and Thr1048) residues are key phosphorylation sites where mutations at these sites prevent phosphorylation and dramatically increase transporter activity.^31^

The other studied heterozygous variants (Arg207His, Gly552Asp and Tyr679Cys) have shown loss of transporter function with normal protein expression.^6 9^ In silico modelling of the Arg207His missense variant disrupted a number of hydrogen bonds between Arg207 and Glu289 and Asp640 and Leu206.^8^ This provides some evidence that these residues may disrupt tertiary protein structure and therefore transporter function and further supports the pathogenicity of the previously reported Glu289Lys, and our family with Asp640Gly and a sensory-ataxic predominant neuropathy.

Despite the extensive work described above, questions regarding *SLC12A6*-related disease remain unanswered. First, how do we corroborate the difference in phenotypes between biallelic and monoallelic forms of the disease? The phenomenon of dominant and recessive disease in the same gene is not uncommon with the archetypal example being the aminoacyl tRNA synthetases. Typically, a biallelic form of disease would be a complex syndrome caused by loss of protein function and a monoallelic form causing CMT through a mechanism other than haploinsufficiency.^4^ However, functional studies in some *SLC12A6* variants causing mono- and bi-allelic disease have demonstrated a loss of transporter function in both, so clearly more complex mechanisms are involved to explain the phenotypic spectrum. Second, why is the phenotypic spectrum of dominant variants so broad and seemingly genotype specific? Distinct genotype-specific differences in phenotype within a gene are reported, with a recent example provided by the *SPTLC1* gene. Most variants, including the UK founder mutation Cys133Trp, cause a HSN through toxic accumulation of abnormal deoxysphinoglipids.^32^ In contrast, specific variants in *SPTLC1* cause juvenile amyotrophic lateral sclerosis via accumulation of canonical sphingolipid products.^33^ Studies have shown a variety of potential mechanisms including loss of KCC3 function through aberrant trafficking, abnormal dimerisation and ion binding, dominant negative effects and gain of transporter function, but none consistently correlate with specific phenotypes.^25 6 9 25^,^27 29^ Kahle *et al* suggested that loss-of-function variants may predominantly affect sensory neurones,and gain-of-function variants motor neurones.^5^ However, the subsequent discovery of Arg207His, that shows no K^+^ flux oocytes, causing a severe sensory and motor neuropathy, argues against this hypothesis.^6^ Finally, how do *SLC12A6* variants cause neuropathy? Original post-mortem studies in ACCPN demonstrated axonal swelling as one of the hallmark pathologies in both the CNS and PNS, and knock-out mice have shown similar findings in sciatic nerves.^19 22^ This finding is easy to rationalise in the context of the function of the co-transporter and its loss-of-function. After demonstrating the first gain-of-function variant, Kahle *et al* proposed a continuum of transporter function, where the extremes cause disease, with gain-of-function variants causing analogous shrinkage, compared with the swelling due to loss-of-function, although this was not demonstrated pathologically. They also proposed altered neuronal excitability due to altered Cl^-^ concentrations as an alternate disease mechanism.^5^ Nerve biopsies in patients with heterozygous Thr991Ala and Tyr679Ser have shown loss of myelinated axons without features of demyelination and loss of myelinated nerve density and thinning of the myelin sheath, respectively, suggesting a predominantly axonal pathology, but some demyelinating features. More histopathological studies are needed to delineate the mechanism of conduction slowing seen in some cases.^5 7^

In conclusion, our study contributes to the existing literature that heterozygous de novo and dominantly inherited variants in *SLC12A6* cause CMT disease. The phenotypic spectrum extends to all known clinical subtypes in CMT and appears to be very genotype specific. The gene should therefore be considered in all forms of suspected inherited neuropathy and included in neuropathy virtual panels in the monoallelic form. Functional studies, including animal models, investigating all the dominantly-acting *SLC12A6* variants are clearly needed to understand disease mechanisms given the possibility that therapeutic approaches will be variant specific.

## Supplementary material

10.1136/jnnp-2025-336643 online supplemental table 1

10.1136/jnnp-2025-336643 online supplemental table 2

10.1136/jnnp-2025-336643 online supplemental table 3

## Data Availability

Data are available upon reasonable request.
