# Supplementary material for: Spectrum of dominant Charcot-Marie-Tooth disease due to SLC12A6 variants
Source: J Neurol Neurosurg Psychiatry. 2026 Jan 7;97(4):e336643. doi: 10.1136/jnnp-2025-336643 (PMC13018852; doi:10.1136/jnnp-2025-336643)
Supplement: online supplemental table 1 [file jnnp-97-4-s001.docx]

| Identifier | S1 | S2 | S3 | S4 |
| --- | --- | --- | --- | --- |
| Variant nucleotide | c.2437-2A>G | c.630G>T | c.1445C>T | c.1217_1218delGGinsTC: |
| Variant amino acid | p.? | p.Trp210Cys | p.Thr482Ile | p.Trp406Phe |
| ACMG Classification | VUS# | VUS | VUS | VUS |
| Phenotype | CMT2 | dHMN | dHMN | CMT2 |
| Ethnicity | White (USA) | Caucasian/white (Belgium) | Caucasian/white (Belgium) | White (USA) |
| Inheritance | AD* | AD* | Sporadic | AD* |
| Age at symptom onset (years) | Teens | 41 | 64 | 45 |
| Age at assessment (years) | 50s | 50s | 60s | 60s |
| Presenting symptom | Muscle twitching, difficulties walking | Walking difficulties | Walking difficulties | Hammertoes, reduced stamina |
| Motor | Mild distal UL and LL weakness and atrophy | Mild distal LL weakness and atrophy | Distal LL atrophy | Mild distal LL weakness and atrophy |
| Sensory | Lenth-dependent mild-moderate large + small fibre sensory abnormalities | Mild large fibre sensory abnormalities in LL | Mild large fibre sensory abnormalities in LL | Mild large + small fibre sensory abnormalities in LL |
| Other features | Romberg’s positive and broad-based gait. CMTES/NS: 14/20 | Nil | Nil | Romberg’s positive, mild tremor, CMTES: 8 |
| Neurophysiology | NCS: Mild-moderate length-dependent axonal sensory and motor neuropathy. EMG: diffuse fibrillation potentials | NCS: mild length-dependant axonal motor neuropathy.  EMG: chronic distal neurogenic changes | NCS: mild length-dependant axonal motor neuropathy.  EMG: chronic distal neurogenic changes | Very limited. NCS: Reduced radial SNAP.  EMG: Distal LL denervation |

**Supplementary Table 1** **Families with single individuals and variants of uncertain significance** *segregation not confirmed, # pathogenic in the recessive form; PVS1 could be applied for this splice variant predicted to create a frameshift variant giving rise to a truncated protein likely to undergo nonsense mediated decay. However, loss of protein product has not been shown to be a mechanism for disease in heterozygous state. CMTES/NS = CMT Examination score/Neuropathy score, dHMN = distal hereditary motor neuropathy, EMG = electromyography, LL = lower limb, NCS = nerve conduction studies, TA = tibialis anterior, UL = upper limb
