# Supplementary material for: Spectrum of dominant Charcot-Marie-Tooth disease due to SLC12A6 variants
Source: J Neurol Neurosurg Psychiatry. 2026 Jan 7;97(4):e336643. doi: 10.1136/jnnp-2025-336643 (PMC13018852; doi:10.1136/jnnp-2025-336643)
Supplement: online supplemental table 2 [file jnnp-97-4-s002.docx]

| **Family** | **F1** | **F2** | **F3** | **F4** | | **F5** | | | | | **F6** | | **F7** | **F8** | **F9** | | **F10** | | | **F11** | **F12** | **F13** |
| --- | --- | --- | --- | --- | --- | --- | --- | --- | --- | --- | --- | --- | --- | --- | --- | --- | --- | --- | --- | --- | --- | --- |
| **Individual** | II.1 | II.1 | II.1 | II.1 | II.2 | I:2 | II:1 | II:2 | II:4 | III:1 | II.2 | III.2 | III.1 | III.1 | II.2 | III.4 | I.2 | II.1 | II.2 | II.1 | II.1 | II.1 |
| **Variant** | R207H | R207H | R207H | M282K | | G286C | | | | | G552R | | G552D | G552D | P569S | | D640G | | | S647P | S647P | T991A |
| **Inheritance** | De novo | Sporadic | De novo | AD | | AD | | | | | AD | | AD* | AD* | AD | | AD | | | De novo | De novo | De novo |
| **Phenotype** | CMT1 | CMT2 | CMTi | CMT^Δ^ | CMT2 | CMT2^a^ | CMTi | CMT2 | CMT2 | CMT2^a^ | CMTi | CMTi | HSN | CMT2 | HMN^b^ | HMN | CMT2-SP | CMT2-SP | CMT2-SP | CMTi | CMTi^+^ | HMN |
| **Ethnicity** | White Brazilian | White British | White USA | English | English | English | English/German | English/German | English/German | English/German | White Aus | White Aus | White USA | Unknown | White Aus | White Aus | White British | White British | White British | White USA | White Italian | White Aus |
| **AAO (years)** | 1^st^ decade | 2-3 | 1^st^ decade | Early teens | Early teens | 40s | 25 | 1st decade | 20 | 13 | 10 | 5 | early 20s | 40 | 34 | 10 | 20s | 30s | Teens | Infancy | Infancy | 1 |
| **AAA (years)** | 20s | 20s | 20s | 60s | 60s | 70s | 50s | 40s | 40s | Teens | 40s | 1^st^ decade | 50s | 50s | 40s | 40s | 70s | 30s | 30s | Teens | 40s | 1^st^ Decade |
| **CMTES/NS** | 18/25 | nd | nd | 26/34 | 19/27 | 21/28 | 15/21 | 9/nd | 6/12 | 5/nd | 24/36 | nd | 6/10 | nd | nd | 8/8 | nd | nd | nd | 10/17 | 23/30 | nd |
| **Presenting symptom** | DW | Falls | Weakness | DW | DW | Tripping | Slow walking | Tripping | Hand cramps | DW | DW/Falls | DW/Falls | Balance | DW | DW | DW | Unsteadiness | Unsteadiness | Unsteadiness | DW/Falls | DW/Falls | Delayed motor skills |
| **Gait** | Steppage | Steppage | Steppage, broad | Wheelchair | Steppage | Steppage | Steppage | Steppage | Mild foot drop | Unknown | Steppage | Normal | Normal | Steppage | Steppage | Steppage | Unknown | Broad-based | Steppage | Steppage, broad | Steppage, broad | Steppage |
| **LL power (dist/prox)** | 0/4 | 1,4/5 | 1/4 | 0/1 | 0/4 | 0/4 | 1/5 | 2/5 | 4/5 | 4/5 | 0/4 | 4/5 | 5/5 | 4/5 | 2-3/4 | 1/4 | 4/5 | 5/5 | 4/5 | 0/5 | 0/4 | 1/5 |
| **UL power (dist/prox)** | 3/4 | 4/5 | 1/5 | 0/3 | 2/4 | 0/5 | 4/5 | 4/5 | 4/5 | 4/5 | 1/5 | 5/5 | 5/5 | 4/5 | 5/5 | 5/5 | 4/5 | 5/5 | 5/5 | 2/5 | 1/4 | 4/4 |
| **Pin prick UL/LL** | Mid-forearm/mid-calf | Normal/Normal | Normal/Knees | Mid forearm/Knees | Normal/Knees | Normal/Mid-thigh | Normal/Mid-foot | Normal/Normal | Normal/Mid-foot | Normal/Normal | Fingers/ knees | Normal/Normal | Normal/Normal | Normal/Normal | Unknown/Normal | Normal/Normal | Mid-forearm/Knees | Normal/Normal | Normal/Normal | Normal/normal | Normal/Ankles | Normal/normal |
| **Vibration UL/LL** | Wrist/Hip | Elbow/Hip | Fingers/Knees | Shoulders/CM | Elbows/CM | Normal/Knee | Normal/Ankle | Normal/Ankles | Normal/Ankles | Normal/Ankles | Elbow/ Knees | Normal/Normal | Normal/Knees | Elbows/Knees | Unknown/Normal | Normal/Normal | Normal/CM | Elbows/Knees | Normal/CM | Normal/normal | Elbows/Knees | Normal/normal |
| **Proprioception UL/LL** | Normal/Ankles | Normal/Normal | Normal/Normal | Fingers/Knees | Normal/Normal | Normal/Ankles | Normal/Normal | Normal/Normal | Normal/Normal | Normal/Normal | Normal/Toes | Normal/Normal | Normal/Ankle | Unknown/Ankles | Unknown/Normal | Normal/Normal | Normal/Knees | Normal/Normal | Normal/Ankles | Normal/normal | Normal/Ankle | Normal/normal |
| **Facial weakness** | Yes | No | No | Yes + myokymia | Myokymia | No | Yes + myokymia | Myokymia | No | No | Yes | No | No | No | Myokymia | Yes | No | No | No | Yes | No | No |
| **Other cranial nerve signs** | No | No | Esotropia | RU | RU, temporalis wasting, ptosis | No | RU | No | No | No | No | No | No | No | No | No | Ptosis | No | No | RU, ptosis | B/l VC palsy, tongue atrophy | No |

**Supplementary Table 2 Clinical features** *segregation not performed, by history, Δ entirely absent action potentials, + low CMAP amplitudes make definitive evaluation of phenotype problematic ^a^ very limited study, ^b^ neurophysiology not seen, AAA = age at assessment, AAO = age at onset, AD = autosomal dominant, Aus = Australian, b/l = bilateral, CM = costal margin, CMT = Charcot-Marie-Tooth disease, CMTES/NS = CMT Examination score/Neuropathy score, DW = difficulty walking, LL (=lower limb) power written as MRC (Medical Research Council) grade out of 5 dist (=distal)/prox (=proximal), where there is different in distal anterior/posterior compartments a comma separates the values, nd = not done, RU = restricted up-gaze, SP- sensory predominant, UL (= upper limb) power as per lower limb, VC = vocal cord, USA United Stated of America
