# Supplementary material for: Spectrum of dominant Charcot-Marie-Tooth disease due to SLC12A6 variants
Source: J Neurol Neurosurg Psychiatry. 2026 Jan 7;97(4):e336643. doi: 10.1136/jnnp-2025-336643 (PMC13018852; doi:10.1136/jnnp-2025-336643)
Supplement: online supplemental table 3 [file jnnp-97-4-s003.docx]

| **Family** | **F1** | **F2** | **F3** | **F4** | | **F5** | | | | | **F6** | | **F7** | **F8** | **F9** | **F10** | | | **F11** | **F12** | **F13** |
| --- | --- | --- | --- | --- | --- | --- | --- | --- | --- | --- | --- | --- | --- | --- | --- | --- | --- | --- | --- | --- | --- |
| **Individual** | II.1 | II.1 | II.1 | II.1 | II.2 | I:2 | II:1 | II:2 | II:4 | III:1 | II.2 | III.2 | III.1 | III.1 | III.4 | I.2 | II.1 | II.2 | II.1 | II.1 | II.1 |
| **Variant** | R207H | R207H | R207H | M282K | | G286C | | | | | G552R | | G552D | G552D | P569S | D640G | | | S647P | S647P | T991A |
| **Phenotype** | CMT1 | CMT2 | CMTi | CMT^Δ^ | CMT2 | CMT2^a^ | CMTi | CMT2 | CMT2 | CMT2^a^ | CMTi | CMTi | HSN | CMT2 | HMN | CMT2-SP | CMT2-SP | CMT2-SP | CMTi | CMTi^+^ | HMN |
| **Age at study (years)** | 20s | Teens | 20s | 60s | 60s | 70s | 50s | 40s | 40s | Teens | 40s | 1^st^ decade | 50s | 50s | 40s | 70s | 30s | 30s | 1^st^ decade | 40s | 1^st^ decade |
| **Median CMAP mV** | 1.1 | 2.9 | 2.0 | absent | 2.6 | absent | 2 | 1.2 | 7.1 | ND | absent | 3.2 | 7.3 | 1.6 | 7.5 | 4.2 | 10.7 | 3.7 | 2.2 | 0.7 | 2.0 |
| **Median CV m/s** | 27 | 41 | 27 | absent | 44 | absent | 33 | 43 | 49 | ND | absent | 40 | 45 | 44 | 61 | 50 | 50 | 45 | 35 | 25 | 28 |
| **Ulnar CMAP mV** | 1.7 | 1.8 | 2.7 | absent | 1.7 | 0.3 | 2.4 | 2.9 | 2.9 | ND | 0.4 | 2.4 | 10.6 | 8.6 | 8.2 | 6.2 | ND | 2.6 | ND | 0.2 | 0.6 |
| **Ulnar CV m/s** | 22 | 54 | 32 | absent | 55 | 29 | 37 | 53 | 44 | ND | 29 | 47 | 48 | 45 | 65 | 50 | ND | 45 | ND | 13 | ND |
| **Peronel (TA) CMAP mV** | absent | 1.4 | 0.1 | absent | absent | ND | 0.3 | 0.8 | 3.1 | ND | 0.4 | ND | 3.7 | 2.1 | absent | 2.4 | ND | 3.2 | ND | absent | ND |
| **Peronel (TA) CV m/s** | absent | 39 | 31 | absent | absent | ND | 46 | 41 | 42 | ND | 25 | ND | 35 | 39 | absent | 44 | ND | 39 | ND | absent | ND |
| **Peroneal (EDB) CMAP mV** | absent | ND | absent | absent | ND | ND | absent | absent | absent | 1.6 | absent | 1.3 | ND | ND | absent | absent | 1.3 | absent | 0.4 | absent | 0.2 |
| **Peroneal (EDB) CV m/s** | absent | ND | absent | absent | ND | ND | absent | absent | absent | 36 | absent | 35 | ND | ND | absent | absent | 49 | absent | 31 | absent | ND |
| **Tibial (AH) CMAP mV** | absent | 0.4 | 0.1 | absent | ND | ND | ND | 0.6 | absent | 3.8 | 0.3 | 9.5 | ND | 0.1 | 0.6 | ND | 6.6 | ND | 2.1 | absent | 2.6 |
| **Tibial (AH) CV m/s** | absent | 27 | ND | absent | ND | ND | ND | 35 | absent | 35 | ND | 35 | ND | 36 | 38 | ND | ND | ND | 31 | absent | 47 |
| **Median SNAP uV** | absent | 1.5 | absent | absent | absent | absent | absent | absent | absent | absent | absent | absent | absent | absent | 12.1 | absent | absent | absent | absent | absent | 22 |
| **Ulnar SNAP uV** | absent | ND | absent | absent | absent | ND | absent | absent | absent | ND | absent | absent | absent | absent | 6.1 | absent | absent | absent | ND | absent | ND |
| **Sural SNAP uV** | absent | absent | absent | absent | ND | ND | absent | absent | absent | ND | absent | ND | absent | absent | 10.8 | absent | absent | absent | absent | absent | 17.9 |
| **EMG** | ND | Chronic denervation | Severe LD chronic denervation | Severe LD chronic denervation | Distal and proximal denervation | ND | Severe distal and proximal denervation | LD chronic denervation + acute denervation | Severe distal and proximal denervation | Distal denervation | Severe chronic denervation distal > proximal | Chronic denervation | Normal | Chronic denervation | Widespread acute and chronic denervation+ CRD | ND | Mild distal denervation in LL | Moderate/severe distal denervation in LL | LD acute and chronic denervation | LD severe acute and chronic denervation | ND |

**Supplementary Table 3 Neurophysiology** Δ entirely absent action potentials, + low CMAP amplitudes make definitive evaluation of phenotype problematic ^a^ very limited study, AH = abductor hallucis, CMAP = compound motor action potential, CRD = complex repetitive discharges, CV = conduction velocity, EDB = extensor digitorum brevis, EMG = electromyography, LD = length-dependant, LL = lower limbs, ND = not done, SNAP = sensory nerve action potential, TA = tibialis anterior
